# Supplementary material for: The Acute Effects of a Fast-Food Meal Versus a Mediterranean Food Meal on the Autonomic Nervous System, Lung Function, and Airway Inflammation: A Randomized Crossover Trial
Source: Nutrients. 2025 Feb 8;17(4):614. doi: 10.3390/nu17040614 (PMC11858349; doi:10.3390/nu17040614)
Supplement: Supplementary file 1 [file nutrients-17-00614-s001.zip › nutrients-3439110-supplementary.pdf]

Supplementary Table S1. Comparison, according to allocation for an intervention order, is Mediterranean and then Fast Food (MdM-FFM) or Fast food followed by a Mediterranean meal (FFM-MdM) during the wash-out period of 7 days

	Total (n=39)	MdM-FFM (n=16)	FFM-MdM (n=23)	*p*‡
**Three-day estimated food record**				
Energy kcal, median [IQR]	2336.1[2134.0; 2554.4]	2204.8[1895.7; 2641.5]	2364.1[2020.4; 2790.5]	0.315‡
Carbohydrates, % TEV, median[IQR]	51.2[48.0;56.1]	51.9[46.7:56.8]	51.0[48.3;54.3]	0.767‡
Protein, g, % TEV, median[IQR]	16.6[14.1;18.4]	15.6[12.4;18.2]	16.6[14.1;18.4	0.358‡
Lipids, g, % TEV, median[IQR]	30.7[26.4;33.0]	32.1[27.5;34.9]	29.6[26.2;33.0]	0.121‡
Saturated, % TEV, median[IQR]	8.2[6.7;9.6]	8.3[6.2;11.3]	8.2[6.8;9.4]	0.525‡
Polyunsaturated, % TEV, median[IQR]	4.3[4.0;5.6]	4.6[3.7;5.8]	4.4[4.1:5.6]	0.944‡
Monounsaturated, % TEV, median[IQR]	9.9[2.7;11.7]	10.1[9.1;12.3]	9.9[8.2;11.7]	0.544‡
Fiber, % TEV, median[IQR]	1.4[1.1;1.8]	1.5[1.1;2.2]	1.5[1.1;1.8]	0.767‡
**Physical activity**				
Pedometer mean number of steps/day (SD)	9648(3146)	9587(2370)	9690(3642)	0.056*
Physical activity (min/day MVPA), median [IQR]	42.2[34.4;63.8]	40.4[32.0:53.8]	49.1[34.4;67.8]	0.288‡
Sedentary activity min/day, mean (SD)	520.4(77.4)	503.4(79.8)	532.4(75.1)	0.724*
Light activity min/day, median [IQR]	272.3[227.3;293.0]	279.1[227.9;304.0]	265.5[227.2;291.5]	0.724‡

*IQR- interquartile range;TEV-total energetic value * Independent sample t test ; ‡Independent samples Mann-Whitney U-test*
